# New generation CAD-CAM materials for implant-supported definitive frameworks fabricated by using subtractive technologies

**DOI:** 10.1155/2022/3074182

**Published:** 2022-03-02

**Authors:** Pinar Cevik, Martin Schimmel, Burak Yilmaz

**Affiliations:** ^1^Department of Prosthodontics, Faculty of Dentistry, Gazi University, Ankara, Turkey; ^2^Department of Reconstructive Dentistry and Gerodontology, University of Bern, Freiburgstrasse 7, 3007 Bern, Switzerland; ^3^Extra muros, Division of Gerodontology and Removable Prosthodontics, University Clinics of Dental Medicine, University of Geneva, Geneva, Switzerland; ^4^Department of Reconstructive Dentistry and Gerodontology, School of Dental Medicine, University of Bern, Bern, Switzerland; ^5^Department of Restorative, Preventive and Pediatric Dentistry, School of Dental Medicine, University of Bern, Bern, Switzerland; ^6^Division of Restorative and Prosthetic Dentistry, The Ohio State University, Ohio, USA

## Abstract

Innovations in digital manufacturing enabled the fabrication of implant-supported fixed dental prostheses (ISFDPs) in a wide variety of recently introduced materials. Computer-aided design and computer-aided manufacturing (CAD-CAM) milling allows the fabrication of ISFDPs with high accuracy by reducing the fabrication steps of large-span frameworks. The longevity of ISFDPs depends on the overall mechanical properties of the framework material including its fit, and the physical properties of the veneering material and its bond with the framework. This comprehensive review summarizes the recent information on millable CAD-CAM framework materials such as pre-sintered soft alloys, fiber-reinforced composite resins, PEEK, and PEKK in high-performance polymer family, and 4Y-TZP. Even though promising results have been obtained with the use of new generation millable CAD-CAM materials for ISFDPs, clinical studies are lacking and future research should focus on the overall performance of these millable materials in both static and dynamic conditions.

## 1. Background

Digital dentistry's influence on patients' experience and dentist's abilities has been remarkable. Computer-aided design and computer-aided manufacturing (CAD-CAM) has become a part of routine dental practice. CAD-CAM can be considered as an umbrella term that includes subtractive or additive manufacturing. In subtractive manufacturing, CAD-CAM systems use milling, drilling, or grinding techniques, which are based on removing or cutting materials from solid blocks or bars. However, dental frameworks are created by adding material in layers in additive manufacturing such as 3D printing or selective laser melting [1].

Conventional fabrication of implant-supported fixed dental prostheses (ISFDPs) has several laboratory steps from spruing to casting [2, 3], which predisposed to human interferences, leading to framework misfit [4]. CAD-CAM technologies eliminate lost-wax casting steps, and rely on complete digital productions, leading to improved accuracy and less cost compared to conventional lost-wax casting techniques [3–7].

The prosthetic material plays an important role in the survival of ISFDPs through stress transmission during chewing cycle, and determines the load-bearing capacity of fixed dental prostheses (FDPs) [8]. The stress generated during function is transferred to implant-framework or implant-bone interface, and can lead to mechanical or biological complications [9]. Screw loosening, peri-implant bone loss, framework fractures, and fractures in prosthetic components are mainly resulted from framework misfit in implant prostheses [9]. Material selection is important for ISFDPs with or without cantilevers, which are subjected to stresses.

Passive fit is important for clinical success and its evaluation for multi-unit frameworks can be done by using 1-screw test, subjective tactile or inspection techniques [9, 10]. Nano-sized framework misfit can occur during pre-fabrication or post-fabrication steps of ISFDPs, and may not be detectable intraorally [9, 11]. Although there is no consensus on the acceptable misfit, 10 to 150 *μ*m misfit was reported to be acceptable in clinical practice for implant frameworks [12, 13].

Published studies on CAD-CAM ISFDPs mostly cover conventional CAD-CAM milling materials such as Ti, zirconia, and Co-Cr. A comprehensive review of recently introduced millable materials, particularly soft alloys and composite resins in high-performance polymer family is lacking in the literature. The aim of this paper was to review and describe recently introduced materials used for the fabrication of ISFDPs by using subtractive CAD-CAM technologies.

### 1.1. Presintered Co-Cr frameworks

Cobalt-chromium (Co-Cr) casting may result in shrinkage of definitive framework [14]. Co-Cr frameworks fabricated by using CAD-CAM can be in presintered soft or sintered hard block forms [15, 16]. Recently introduced presintered soft alloys are also known as soft-milled alloys [17].

Conventional Co-Cr alloys include 55–63 wt% cobalt and 25–28 wt% chromium with a melting point of >1490°C [16]. Soft-milled alloys and their ion compositions may vary across different manufacturers. Resistance to ion release is a highly important factor in terms of biocompatibility. Released substances from the material could trigger a biological reaction, thus material corrosion could be harmful to adjacent tissues. Kassapidou et al. [18] reported that cast alloy had the highest total ion release when compared with hard milling Co-Cr, soft-milled Co-Cr, and laser-sintered Co-Cr.

The current brands of soft-milled alloys in different compositions are listed in Table 1. A presintered soft block can be milled by dry machining with low contamination risk, which is an easier process than sintered Co-Cr milling [19]. The milled framework should be sintered in an argon atmosphere sintering oven at 1250-1400°C for 1 hour or 2 hours [20, 21]. While there is no contraction shrinkage of a fully-sintered alloy used for hard milling, the shrinkage rate is 10-11% for soft-milled alloys after sintering [14, 22, 23]. The post-sintering after milling could affect the mechanical strength of soft-milled alloy [21], and 1350°C was reported to be the most suitable temperature [20, 21].

Favorable marginal fit for single crowns fabricated by using soft-milled Co-Cr, in some situations, outperforming cast crowns was reported [24, 25]. However, the results of studies should be carefully interpreted considering the differences between single crown and complete-arch fabrication methods, and the size of the restoration [26, 27]. Daou et al. [28] investigated the marginal accuracy of 3-unit FDPs fabricated with soft-milled alloy, casting Co-Cr alloy, or presintered zirconia. The marginal discrepancy of restorations in all groups was similar and within the clinically acceptable range (<120 *μ*m). A similar study [17] reported that 3-unit FDPs fabricated by using presintered soft alloy showed marginal discrepancy (below 100 *μ*m) similar to pre-sintered zirconia or cast Co-Cr alloy. Izadi et al. [29] investigated 3-dimensional (3D) changes in cast or soft-milled 5-unit implant frameworks. They found dimensional changes with both cast and soft-milled frameworks, while the difference between was not significant. In addition, soft-milled alloys were reported to have no advantages over cast alloys in reducing transverse changes of frameworks. This means that when the abutments are located in a curve, transverse changes and back and forth movement would potentially remain as a problem in the soft-milled framework [29].

The geometric discrepancies of soft-milled complete-arch implant-supported fixed dental prostheses (CAISFDPs) frameworks were compared [14]. The horizontal and angular discrepancies of frameworks varied depending on the alloy manufacturer while internal discrepancy did not. Taşın et al. [22] reported that hard milling had the lowest vertical misfit compared with soft milling and direct metal laser sintering. Woo et al. [30] reported that soft-milled and hard-milled CAISFDPs had similar marginal accuracy (<120 *μ*m) compared with cast, and recommended soft-milled Co-Cr alloy for CAISFDPs.

Co-Cr frameworks can be veneered with polymethyl methacrylate (PMMA) [22, 31], composite resin [32], or porcelain [15, 23, 33, 34]. Li et al. [16] indicated that soft-milled Co-Cr had superior strength after repeated porcelain firings when compared with hard-milled or cast Co-Cr. Lee et al. [23] also stated that the bonding ability of soft-milled alloys to porcelain was similar to that of cast alloy.

Aside from the abovementioned reports, studies on the implementation of soft milling techniques for CAISFDPs are scarce, and further data is required for their long-term use. To the best of the authors' knowledge, previous publications provide no information on the load-bearing capacity of soft-milled alloys, and no consensus has been reached on their marginal fit.

### 1.2. Fiber-Reinforced Composite Resins

New generation CAD-CAM fiber-reinforced composite resins (FRCs) have a high concentration of multidirectional interlacing of high glass fibers when compared with conventional FRCs, and have been used as ISFDP frameworks because of their claimed shock-absorbing behavior, low elastic modulus, and high flexural strength [35–37]. FRCs have superior optical properties compared with metal frameworks [36]. The most common FRCs are listed in Table 2.

A metal framework may transfer masticatory forces directly onto the bone surface due to its rigid structure [36]. However, having low elastic modulus, FRC frameworks may absorb the energy from the masticatory cycle, providing the advantage of reduced stress on the peri-implant bone surface [36, 38]. Previous reports demonstrated that FRC frameworks led to less stress on cancellous and cortical bone when compared with that generated by metal frameworks [36].

A recently introduced FRC (Trinia; Shofu Dental Corp) consists of 60% multidirectional interlacings of glass fibers and 40% epoxy resin. Trinia is nonflammable, biocompatible, and lightweight [39], and has been used as a framework for ISFDPs due to its high bending and compressive strength [39, 40]. Because Trinia has an anisotropic nature, the mechanical properties of the framework may differ by fiberglass orientation [41, 42]. It was reported that load applied to the fiberglass along different axes could affect flexural strength [41]. Suzaki et al. [37] reported that milled Trinia, where fiberglasses were longitudinally parallel to the bars had high flexural strength.

Fiberglass in Trinia frameworks tends to be soluble [43], and its exposure can be irritating to soft tissues [40]. Therefore, frameworks should be veneered to cover the exposed fibers [43]. FRC can be veneered with composite [36, 39, 44], acrylic resin [38, 45], and porcelain crowns can be bonded on.

Ewers et al. [46] reported the use of 101 Trinia frameworks for the fabrication of multi-unit FDPs or CAISFDPs. Sixteen patients received mandibular CAISFDPs on 4 short implants with a prosthetic success rate of 100% in 64 months. Only 1 FDP framework broke while there was no chipping for any prostheses. Trinia was reported as a promising alternative to metal ISFDPs [46]. Biris et al. [39, 40] reported the successful application of FRC (Trinia) frameworks for ISFDPs. After 18 months, 24 patients who received 35 implants expressed high satisfaction, and there was no deformation at implant or framework level [39]. They also reported the 30-month follow-up of 35 ISFDPs on 42 implants with promising biological and mechanical outcomes [40].

The survival probability of 3-unit implant-supported FRC frameworks (Trinia) was not statistically different between 3 mm^2^ or 12 mm^2^ connector areas [44]. It was also stated that fatigue analysis of FRC frameworks revealed favorable results similar to metal frameworks [44].

Load at failure and fatigue failure values of 3-unit implant-supported composite veneered FRC (Trinia) frameworks were evaluated [47]. The FRC frameworks fractured at 1679.56 N, while for composite resin veneered zirconia, the fracture occurred at 1905.47 N. After fatigue testing [47], veneering composite resin fractures were observed at the cervical of FRC frameworks due to tensile stresses rather than compressive (occlusal area). The favorable load-bearing values observed in FRC (Trinia) frameworks maybe because the elastic modulus of veneering composite resin is close to that of the FRC. In another study, load to fracture value of FRC (Trinia) frameworks with a 10-mm cantilever was 2036.69 N, where this value was 5800.81 N for 3Y-TZP zirconia [35].

The photoelastic stress analysis of FRC (Trinia), Co-Cr, and Ti frameworks for mandibular CAISFDPs was evaluated [45]. FRC frameworks exhibited the most favorable results including reduced weight and shock absorption when compared with Co-Cr and Ti bars. FRC frameworks also generated less stress in the cervical region on the implants, while more rigid Co-Cr and Ti frameworks resulted in increased stress in the cervical region of implants [45]. Another study [48] reported the early results of FRC (Trinia) on 4 short implants. None of the frameworks had fractures in an average of 19.5 months. Forty implants were placed, and their survival rate was 97%. The cantilever lengths were measured by computing bridge span to anteroposterior implant span ratio which was up to 5.7. The authors claimed that longer cantilevers could be successfully used in FRC CAISFDPs without technical or biological complications [48]. Seemann et al. [49] later reported the mid-term outcomes of those FRC CAISFPDs, and stated that these dentures provided cost-effective, safe, stable solutions with favorable biological outcomes for severely atrophic mandible. The cumulative 5-year implant survival rate was 98%, and the average marginal bone loss around distal and mesial implants was 0.17 mm, within the physiological range [49]. The prosthetic survival rate was 100% after a 4-year follow-up, while a 27.8 mm distal cantilever fractured after 4.8 years of loading. Passaretti et al. [38] reported the application of FRC (Trinia) frameworks for mandibular cement-retained CAISFDPs on 4 short implants. The maximum cantilever length was 21 mm and the opposing dentition was removable complete denture. The authors stated that FRC CAISFDPs veneered with acrylic resin provided cost-effective results with good esthetics and high patient satisfaction. Wagner et al. [50] used FRC (Trinia) frameworks for the fabrication of maxillary cement-retained CAISFDPs on 4 short implants for 18 patients. At 1 year, the cumulative survi.val rate of 72 implants was 97.2%, and the prosthetic survival rate was 100%. The opposing dentitions were natural teeth, partial dentures, or ISFDPs. The marginal bone level ranged from 0.4 to 1.3 mm (average) [50], and was comparable to previous studies reporting 0.74 to 1.41 mm marginal bone loss in 1 year [51, 52]. It was also reported that that new generation FRC (Trinia) frameworks with high-impact acrylic superstructure could successfully be integrated into the all-on-4 concept with predictable treatment outcomes [53].

Trilor (Bioloren) is a recent high-tech CAD-CAM FRC, which consists of multidirectional integrated fiber glasses in an epoxy resin matrix [35]. Trilor can be dry- or wet-milled for the fabrication of single, multi-unit, or CAISFDPs frameworks [54], and can be veneered by using composite resin, acrylic resin, or bonded to lithium disilicate crowns [54, 55].

Regarding the mechanical properties of FRC resins, millling direction in different angulations resulted in different flexural strength and surface roughness for FRC (Trilor) [56]. When the milling direction changed from vertical to diagonal, it was reported that the flexural strength of FRC (Trilor) specimens was 3 times higher. Additionally, the load-to-failure values for FRC (Trilor) frameworks with a 10-mm cantilever with or without Ti-base, and Trilor specimens without Ti-base had higher load-to-failure values (2817.05 N) than those of Trinia, PEEK, modified PEEK, or PEKK. Trilor specimens with Ti-base had higher load-to-failure values compared with Trinia with Ti-bases [35].

To the best of authors' knowledge, there are no reports on marginal fit or the load-bearing capacity of new generation FRC (Trinia and Trilor) CAISFDPs.

### 1.3. PAEK

PAEK (Polyaryl ether ketones) materials, commonly referred to as PEEK (Polyetheretherketone) or PEKK (Polyetherketoneketone), belong to the class of engineering polymers, [57] and have been introduced as alternatives to metal alloy and zirconia for implant frameworks. PEEK (Polyetheretherketone) is a thermoplastic semi-crystalline high-performance polymer. Noncorrosive, nonconductive properties, radiolucency, high-temperature stability, low plaque affinity, low weight, resistance to water absorption, and high biocompatibility make PEEK a favorable dental material [58, 59]. However, like other polymers, fumes resulted from overheating of PEEK can be harmful [57]. The biofilm formation on PAEK implant structures were lower than that on zirconia or Ti implant components [60, 61]. Mucosal response to PEEK as a prosthodontic material was good [60]. The commercially available PAEK materials are listed in Table 3.

Pigments, titanium dioxide, or ceramic fillers have been added to PEEK for its modification and a modified PEEK has been obtained [62]. A recent modified PEEK, which was obtained incorporating 20% nano-ceramic fillers (BioHPP) into non-filled PEEK, is claimed to be more resistant than PEEK, and can be used with implants due to the consistent homogeneity of the structure [63]. PEEK frameworks can be veneered with composite resin [8, 59, 62, 64–66], polymethyl methacrylate resin (PMMA) [67, 68], high-impact PMMA veneers [65], or bonded to lithium disilicate crowns [69]. Veneering composite resin or acrylic resin could be an alternative to porcelain due to their low weight and easy reparability, which is important for long-span frameworks. The composite resin was found to have better esthetics and color stability than acrylic resin [59].

Studies, which evaluated the marginal gap of single-unit PEEK frameworks reported that their marginal gaps were not clinically acceptable [70, 71]. On the contrary, Attia et al. [72] reported that the marginal gap of PEEK single-unit restorations was clinically acceptable (<120 *μ*m). Only a few reports are available on the use of PEEK for CAISFDPs. Zoidis [65] stated that unlike metal frameworks, PEEK cannot be sectioned and welded in case of misfit. Therefore, the use of a transfer index was recommended to verify implant positions and to ensure the framework fit [65].

Short-term outcomes were reported for acrylic resin veneered PEEK CAISFDPs on 4 implants in 37 patients [67]. One-unit cantilevers or no distal cantilevers were used. The marginal bone loss was 0.37 mm in 1 year. Screw loosening was observed in one prosthesis with one-unit cantilever and in two prostheses without cantilever. Longer distal cantilevers resulted in veneer fractures, potentially because PEEK framework was flexing when cantilevers were long. Considering its biological integrity, high patient satisfaction, and low marginal bone loss, PEEK was reported as a favorable option for CAISFDPs. Three-year follow-up of the same study, where a total of 49 CAISFDPs were evaluated reported an average marginal bone loss of 0.4 mm [68]. Low marginal bone loss was attributed to the shock-absorbing ability of PEEK frameworks.

Cabello-Domínguez [69] et al. reported a 3-year follow-up of a completely edentulous patient rehabilitated with maxillary zirconia prosthesis and a mandibular PEEK framework with lithium disilicate crowns and layered composite resin. In 3 years, there were 60% and 50% marginal bone loss around implants loaded with PEEK and zirconia frameworks, respectively. AL-Rabab'ah [64] used modified PEEK CAISFDP frameworks with pre-manufactured composite resin superstructures and reported minimal to no bone loss around implants with no complications in 1 year [64].

The load-bearing capacity of anatomic 3-unit, non-veneered, modified PEEK frameworks was investigated and the mean fracture load was 2354 N [73]. A previous study reported the mean fracture load of 3-unit, non-veneered modified PEEK specimens with a flat surface as 1,383 N [74]. Stawarczyk et al. [66] investigated the load-bearing capacity of anatomic 3-unit composite resin veneered modified PEEK restorations, and surface pretreatments, primer, or composite resin type did not affect the results. Nazari et al. [8] reported 1430 N as the fracture load for anatomic 3-unit and resin composite veneered modified PEEK specimens, which was 1518 N in Jin et al. ‘s [59] study. PEEK frameworks required increased connector area to increase fracture loads [59]. The performance of PEEK CAISFDPs under loads is not available in the literature, to the best of authors' knowledge.

An increase in strength but a decrease in elastic modulus of framework could lead to increased maximum load-to-fracture values for different cantilever lengths in CAISFDPs [75]. Load-to-failure values for different CAD-CAM frameworks with a 10-mm cantilever ranged between 2021-2159 N for 100% PEEK values were 2000 N for modified PEEK frameworks [35]. The biomechanical behavior of different frameworks with 10-mm cantilevers used in CAISFDPs was reported in a 3D FEA study [76]. PEEK frameworks had high stress concentration at the bone-framework interface, which was above clinical acceptance level of 5 MPa. PEEK frameworks had reduced stress concentration on the framework material, abutment, and implants, but an increased stress concentration on the trabecular bone. On the contrary, Co-Cr, Ti, and zirconia had increased stress values in the framework material, but low-stress values on the trabecular bone [76].

PEKK was introduced as a lightweight framework material, and has good biological properties [77]. PEKK had a lower inflammatory response than PMMA in an animal study [78], and can be used for the rehabilitation of patients with metal allergies [77]. PEKK consists of titanium dioxide particles by 20% and an additional ketone group, which lead to [79] 80% higher compressive strength and better fatigue properties than PEEK [72, 80, 81]. Having shown promising performance, PEKK could be a suitable permanent framework material for high-stress bearing areas [82, 83]. Han et al. [84] reported the use of PEKK for a maxillary CAISFDP and a partially edentulous mandible. PEKK frameworks had passive fit, and PEKK's performance was promising with no pathogenic signs or prosthetic complications in the short-term [84]. Due to its low elastic modulus, PEKK was reported to generate low stresses at the terminal abutment of the framework, providing the advantage of possible shock absorption and stress distribution [84]. PEKK was also used for a cement-retained CAISFDP with acrylic resin crowns [85]. The esthetic and the functional outcomes were favorable at 1-year follow up.

PEKK requires veneering due to its greyish color and can be veneered with ceramic [82–84], composite resin [80, 82, 84, 86] or acrylic resin [80, 84, 87]. PEKK can be used as a framework either in cement-retained [85] or screw-retained CAISFDPs. Acrylic resin [85] or ceramic [80] crowns can also be bonded to PEKK frameworks. It was reported that rigid frameworks veneered with acrylic resin provide shock absorption because of the low modulus of the veneering material [65, 88, 89]. Accordingly, flexible frameworks veneered with low modulus composite resin or acrylic resin rather than porcelain may result in favorable stress distribution [48, 65]. However, load-to-fracture values of anatomic 3-unit PEKK frameworks with lithium disilicate crowns were found higher (1526 N) than PEKK veneered with composite resin (1069 N) [82]. It was stated that the combination of PEKK framework and high-strength veneering glass-ceramic could be used for the ISFDPs in the posterior region [82].

The marginal fit of non-anatomic single-unit PEKK frameworks [90] was better compared with zirconia. Anatomic single-unit composite resin veneered PEKK restorations had favorable fatigue life and limit (790 N) after cyclic loading [79]. The load-bearing capacity of anatomic single-unit monolithic PEKK frameworks was 2037 [83], and it was stated that PEKK could be used as a definitive monolithic posterior crown because of its mechanical properties [83]. Mean load-to-fracture value of 10 mm-cantilevered non-veneered PEKK material was reported as 1889 N [35].

Lee et al. [86] demonstrated 3D finite element analysis (FEA) of PEKK, Ti, or zirconia frameworks for the fabrication of CAISFDPs. They found that PEKK generated less stress on the framework itself while more stress on the veneering material, due to its low-elastic modulus and flexibility [86]. The shock-absorbing feature of PEKK frameworks was found low and limited on the increased compressive stress areas whereas, in the tensile stress areas, stress transferred to the implant and adjacent tissues were high. In addition, PEKK frameworks had larger bending movements, leading to increased bending forces on the implants when compared with rigid Ti or zirconia frameworks [86]. The authors speculated that the use of PEKK as a definitive framework in CAISFDPs was questionable [86]. However, these results were based upon data from an FEA, and there is no agreement that PEKK frameworks generate more stress on the bone.

Published studies on PEKK are limited to case reports [77, 80, 84, 85] or FEAs [77, 86, 90], and fail to report the load-bearing capacity or marginal misfit of the PEKK in CAISFDPs. Further studies are needed to better indicate the material's behavior over long-term clinical use.

### 1.4. 4Y-TZP –Cubic Zirconia

Polycrystalline zirconia has been in use for the fabrication of crowns, short- and long-span bridges, CAISFDPs, and implants [91]. Tetragonal partially stabilized zirconia, 3Y-TZP, contains 3 mol % Y_2_O_3_. The first and the second generations of 3Y-TZP have high flexural strength (>1,000 MPa). Recent advances in optical and mechanical properties of highly opaque 3Y-TZP have led to the innovation of new generations with higher translucency. For the 3^rd^ and 4^th^generations, first, attempts were made to increase Y_2_O_3_ content to 5 mol % (5Y-TZP). Increasing yttrium content to 5 mol % resulted in high translucency with reduced mechanical properties after artificial aging with mechanical cyclic loading [92]. It was reported that increasing the yttrium content to 5 mol% resulted in reduced fracture strength of single-unit restorations, however, the fracture strength still exceeded 3000 N after artificial aging with cyclic loading [92]. Later, the Y_2_O_3_ content was decreased to 4 mol% [93–95], and an optically isotropic material, also known as 4Y-TZP was obtained having a stable ratio of cubic and tetragonal zirconium oxide polycrystals [94, 96]. This 4^th^ generation of zirconia is a balanced material in terms of strength and translucency, being between 3Y-TZP and 5Y-TZP [92, 97]. 4Y-TZP is used from single-unit to complete-arch prostheses [97–99] in monolithic or multilayered form for long-span tooth- or ISFDPs up to 14 units with a maximum of two pontics [99]. Commercially available 4Y-TZP materials are listed in Table 4.

CAD-CAM zirconia has been in use in-office and can be milled from soft blocks and sintered. Standard sintering takes 4-12 h to complete at 1450°C, speed sintering takes place at 1510°C for 30-120 minutes, and high-speed sintering requires less than 30 minutes at temperatures around 1580°C [100]. Studies reported that high-speed sintering may affect the mechanical properties of 4Y-TZP specimens. In this regard, Michailova et al. [97] reported that high-speed sintering did not negatively affect the fracture load of 4Y-TZP specimens. In addition, other studies reported that high-speed sintering resulted in similar or higher fracture loads than that of conventional sintering [95, 97, 100, 101].

Zacher et al. [102] investigated the fatigue failures of 3-unit screw-retained 4Y-TZP specimens as 2129.3 N, while cement-retained specimens fractured at 2094.3 N in their study. The load-to-failure values of 10-mm cantilevered 4Y-TZP with or without Ti-base were evaluated [35]. The 4Y-TZP specimens without Ti-base exhibited higher load-to-failure value (3506.09 N) than PEEK, modified PEEK, PEKK, FRC (Trilor), and FRC (Trinia). The 4Y-TZP specimens had lower load-to-failure values than those of 3Y-TZP specimens, and it was speculated that cubic 4Y-TZP may be an alternative to 3Y-TZP zirconia for cantilevered ISFDPs.

There are no recent publications, which reported the mechanical strength of 4Y-TZP zirconia as a framework material for CAISFDPs. Future research should investigate 4Y-TZP's *in vitro* and clinical performance when used for the fabrication of ISFDPs.

## 2. Conclusions

There is considerable interest in recently introduced millable CAD-CAM materials for the fabrication of ISFDPs and promising results have been published for their in vitro and short-term clinical performance. Clinical studies in the short- or mid-term use of PEEK or FRC (Trinia) frameworks reported high patient satisfaction, biological integrity, and low mechanical or prosthetic complications on their use for the CAISFDPs. In addition, the use of PEEK or FRC (Trinia) frameworks in CAISFDPs reported low to average marginal bone loss, which was attributed to shock-absorption abilities of the materials. On the other hand, studies regarding the use of PEKK in CAISFDPs are limited to case reports, and report neither the marginal gap of the frameworks nor the marginal bone loss. In addition, there is no data available in the clinical use of 4Y-TZP CAISDPs frameworks. Furthermore, soft-milled alloys used in the CAISFDPs seem to have promising results regarding the marginal accuracy according to *in vitro* studies. Nevertheless, no consensus has been reached on the marginal accuracy of soft-milled alloys, and further data is necessary for their clinical use.

In summary, clinical studies are needed to corroborate the performance of PAEKs, pre-sintered soft-milled alloys, FRC resins, and 4Y-TZP in the short-term, and additional information on their long-term behavior. Furthermore, future research on mechanical and physical properties of recently introduced materials primarily in dynamic conditions is required.

## Figures and Tables

**Table 1:** Commercially available soft milling alloys.

Material	Manufacturer	Composition	Elastic modulus	Tensile Strength	Flexural Strength
Ceramill Sintron	AmannGirrbach,Koblach, Austria	Co 66, Cr 28,Mo 5, Si<1, Fe<1, Mn<1	200 GPa	900 MPa	
Sintermetall	Zirkonzahn, South Tyrol,Italy	Co 65, Cr 27,Mo 5, C, N <1			1.243 MPa
Soft Metal	LHK, Daegu,Chilgok, Korea	Co 63.4, Cr 29,Mo 5.8, Si 0.8,other elements <1	237 GPa [103]		

**Table 2:** New generation commercially available FRC framework materials.

FRC material	Elastic modulus	Tensile strength	Flexural strength	Composition	Connector Cross-section	Cantilever
Trinia (Shofu Dental Corporation, San Marcos, California)	18.8 GPa	169 MPa	393 MPa	Glass Fabric Prepreg,A multidirectional interlacing of fiberglass in several layers: 50-60%wt; epoxy resin: 40-50%wt	Minimum 7.0mm^2^ connector	Maximum 15 mm cantilever
Trilor (Bioloren S.r.l, Saronno,Italy)	26 GPa	380 MPa	540 MPa	High-performance techno-polymer matrix with multi-directional glass fiber reinforcement; made up of about 74%wt of glass fibers	7.0 mm^2^	1 pontic

**Table 3:** Commercially used PAEK materials.

Material	Elastic modulus	Tensile strength	Flexural strength	Composition	Connector Cross-section	Cantilever
PEEK-Juvora (Juvora Ltd, Wyre,Lancashire, UK)	4.1 GPa	115 MPa	164 MPa	100% PEEK	12 mm^2^	Max-1 pontic
PEEK-Coprapeek (White Peaks Dental SystemsGmbH & Co. KG., Essen,Germany)	—	—	186.6 MPa	100% PEEK	10mm^2^-anterior 16mm^2^-posterior	Max-2 pontics
Modified PEEK-BreCamBioHPP (Bredent GmbH & Co. KG.,Senden, Germany)	4.62 GPa	97 MPa	>150 MPa	80% PEEK, 20% ceramic filler	13 mm^2^	Max-1 pontic
PEKK-Pekkton Ivory (Cendres Métaux SA, Biel/Bienne, Switzerland	5.1 GPa	115 MPa	200 MPa	80% PEKK 20% TiO_2_	12mm^2^-anterior 14mm^2^-posterior	Max-2 pontics
Modifed PEEK-Dentokeep- (Dentokeep PEEK Disc, nttrading,Karlsruhe, Germany)	>3.8 GPa	—	190 MPa	80% PEEK 20%TiO_2_	10 mm2-anterior 16 mm2-posterior	—
Modified PEEK-Vestakeep (Vestakeep, EvonikIndustries	4-5.1 GPa	110 MPa	165-178 MPa	80% PEEK 20% TiO_2_		—
Modified-PEEK-White color, (Tecno Med Mineral, Zirkohnzahn S.r.l, Gais, Italy)	4.1 GPa	—	201 MPa	High performance resin, special ceramic reinforcement PEEK	13 mm^2^	Max-2 pontics
PEEK- DD peek MED (Dental Direkt GmbH, Spenge, Germany)	≥3,8 GPa	—	≥155 MPa	99% PEEK 0.01% additives		
Bone	14 GPa	104–121 MPa		—		na
Dentin	15 GPa	104 MPa		—		na

**Table 4:** Commercially available Cubic zirconia (4Y-TZP) materials.

4Y-TZP Material	Elastic modulus	Sintering temperature	Flexural strength	Composition	Connector Cross-section	Cantilever
DD cube ONE (Dental Direkt GmbH, Spenge, Germany)	>200 GPa	1450°C	>1250 MPa	ZrO₂ + HfO₂ + Y₂O₃ ≥99,0; Y₂O₃ <8; Al₂O₃ <0,15; Other oxides<1,0%		maximum of two teeth per pontic span
Ceramill Zolid HT+ (Amann GirrbachAG, Koblach, Austria)	≥200 GPa	1450°C	1100 +/-150 MPa	ZrO_2_ + HfO_2_ + Y_2_O_3_: ≥99.0 Y_2_O_3_: 6,7 - 7,2 HfO_2_: ≤5 Al_2_O_3_: ≤0.5 Other oxides: ≤1%	> 12 mm^2^	1-pontic for
Pritidenta (priti®multidisc ZrO2 multicolor Extra Translucent)	210 GPa		> 1,150 MPa (Bridges up to 16 pontics)	ZrO_2_/HfO_2_: 92.1-92.65% Y_2_O_3_: 6.65-7.95% Al_2_O_3_: < 0.4% Other oxide:< 0.7%	6mm^2^,anterior 9mm^2^, posterior	-Minumum framework thiknesses: 0.6 mm
Katana (Katana Zirconia ML-HT Disc, NORITAKE CO, LIMITED)		1500°C	1125 MPa	—	12 mm^2^ or more for cantilever; 9mm^2^or more for posterior pontics	1 pontic
IPS e.max ZirCAD MT BL Multi4, Ivoclar-Vivadent AG, Schaan, Liechtenstein		1600°C	Biaxial flexural strength: 850 MPa	ZrO_2_:86.0 – 93.5% Y_2_O_3_: > 6.5% ≤ 8.0% HfO_2_ ≤5.0% Al_2_O_3_ ≤1.0% and other oxides ≤1.0%		

## Additional Points

*Clinical Significance*. CAD-CAM soft Cr-Co alloys, fiber-reinforced composite resins, PEEK, and PEKK in high-performance polymer family, and 4Y-TZP are current alternatives to cast metal alloys and 3Y-TZP zirconia for the fabrication of ISFDP frameworks. These materials provide promising functional and esthetic outcomes in the short term when used for the fabrication of ISFDPs. However, clinical studies are needed to corroborate their short-term performance.
